# Physiological Farming

**Published:** 1860-12

**Authors:** 


### PHYSIOLOGICAL FARMING.

Michael Sullivant owns, and has paid for, one hundred thousand acres of land, near Homer, Illinois. He keeps a large amount of money always on hand, to pay his workmen at the end of every week. They take their breakfast at half-past five o'clock every morning, and ride to the place of work. They dine at noon, their dinners being carried to them. They quit work at sundown, and ride home. In this short narration, there is a knowledge of physiology, of body and of mind, which is highly creditable to the Napoleon of farmers. To show the wisdom of the whole arrangement, would require a commentary of many pages. To remark in brief, profitably:

The land having been paid for, saves an immense amount of up-hill, hard, dragging work. To have to pay out any considerable part of farm earnings for eating interest, even at six per cent, and every once in a while, an installment of the purchase-money, instead of being able to expend these in substantial improvements, in the purchase of fertilizers and labor-saving machines, such as McCormick's reaper, the grain-sower and the steam plow, make the cultivation of the soil, in innumerable cases, a literal galley-life. By this same course, many an honest, industrious, and ambitious farmer has worked himself to death, before he lived out half his days.

To "farm" pleasantly and successfully, not only must the land be paid for, but a liberal amount of money should be on hand to meet emergencies, and pay the laborers always, promptly, in full, at the hour, and to the last cent, in money. Such a course will always secure the best hands, and at the lowest prices; and more, it keeps the men; thus avoiding unprofitable and troublesome changes; and if unavoidable circumstances compel them to leave, they leave with a kindly feeling, and will not fail to recommend others to fill their places; hence, such a farmer is every where spoken well of, at all times has his pick and choice of hands, and more, he will get more work out of his men; for knowing that their pay is always in full, and at the hour, they have the strongest stimulus to make an effort to retain their places, and to do their work well; and by doing it cheerfully, they perform more work in a given time.

They take their breakfast early in the morning before they go to their work, thus preventing the inevitable exhaustion which results from working several hours on an empty stomach; and besides, largely contributing to the prevention of fever and ague, by fortifying the stomach against the morning miasma, which always abounds in flat and fertile countries, causing innumerable cases of chills, fever, diarrheas and dysenteries.

Another little item merits attention. The workmen are not allowed to waste their early strength by walking a mile or more to their places of labor; means are provided for riding there, so as to enable them to commence the day's work with the full stock of strength secured by the rest of the night. This same husbanding of the energies against useless waste is looked to in having their dinners carried to the workmen; and then, when quite enough wearied by the legitimate labors of the day, they ride home so as to prevent that exhaustion, that over-fatigue, which a walk of a mile or two or three, would otherwise occasion, and which would have to be subtracted from the strength of the next day. For let all remember, that the overwork of to-day is but a draft on to-morrow, which "must be paid," infallibly, and to the utmost farthing.

It is pitiful to think of unrequited toil; of unavailing labor; of squandered strength; of wasting, wearing care, and of the unsuccessful lives, which every year witnesses, by the unwisdom of men in owning more land than they have paid for, or can thoroughly and easily cultivate.

In any given case, a man who has paid for his ten-acre farm, and always has money enough on hand, without owing a dollar, to pay his workmen, and to take advantage of passing circumstances, will live longer, more happily, more usefully and successfully, than his neighbor, who works harder by many-fold, in cultivating ten times the amount of land, yet unpaid for, who is always "behind hand" with his laborers, and pressed for money.

